# Association between IgM Anti-Herpes Simplex Virus and Plasma Amyloid-Beta Levels

**DOI:** 10.1371/journal.pone.0029480

**Published:** 2011-12-28

**Authors:** Catherine Féart, Catherine Helmer, Hervé Fleury, Yannick Béjot, Karen Ritchie, Philippe Amouyel, Susanna Schraen-Maschke, Luc Buée, Jean-Charles Lambert, Luc Letenneur, Jean-François Dartigues

**Affiliations:** 1 INSERM, U897, Bordeaux, France; 2 Université Bordeaux Segalen, Bordeaux, France; 3 Department of Neurology, University Hospital of Dijon, Dijon Stroke Registry, EA4184, Faculty of Medicine of Dijon, University of Burgundy, Dijon, France; 4 INSERM, U888, Montpellier, France; 5 Faculty of Medicine, Imperial College London, London, United Kingdom; 6 INSERM, UMR744, Lille, France; 7 Institut Pasteur de Lille, University of Lille 2, Lille, France; 8 INSERM, U837, Lille, France; London School of Hygiene and Tropical Medicine, United Kingdom

## Abstract

**Objective:**

Herpes simplex virus (HSV) reactivation has been identified as a possible risk factor for Alzheimer's disease (AD) and plasma amyloid-beta (Aβ) levels might be considered as possible biomarkers of the risk of AD. The aim of our study was to investigate the association between anti-HSV antibodies and plasma Aβ levels.

**Methods:**

The study sample consisted of 1222 subjects (73.9 y in mean) from the Three-City cohort. IgM and IgG anti-HSV antibodies were quantified using an ELISA kit, and plasma levels of Aβ_1–40_ and Aβ_1–42_ were measured using an xMAP-based assay technology. Cross-sectional analyses of the associations between anti-HSV antibodies and plasma Aβ levels were performed by multi-linear regression.

**Results:**

After adjustment for study center, age, sex, education, and apolipoprotein E-e4 polymorphism, plasma Aβ_1–42_ and Aβ_1–40_ levels were specifically inversely associated with anti-HSV IgM levels (β = −20.7, *P* = 0.001 and β = −92.4, *P* = 0.007, respectively). In a sub-sample with information on *CLU*- and *CR1*-linked SNPs genotyping (n = 754), additional adjustment for *CR1* or *CLU* markers did not modify these associations (adjustment for *CR1* rs6656401, β = −25.6, *P* = 0.002 for Aβ_1–42_ and β = −132.7, *P* = 0.002 for Aβ_1–40;_ adjustment for *CLU* rs2279590, β = −25.6, *P* = 0.002 for Aβ_1–42_ and β = −134.8, *P* = 0.002 for Aβ_1–40_). No association between the plasma Aβ_1–42_-to-Aβ_1–40_ ratio and anti-HSV IgM or IgG were evidenced.

**Conclusion:**

High anti-HSV IgM levels, markers of HSV reactivation, are associated with lower plasma Aβ_1–40_ and Aβ_1–42_ levels, which suggest a possible involvement of the virus in the alterations of the APP processing and potentially in the pathogenesis of AD in human.

## Introduction

Previous researches have suggested that Herpes Simplex Virus (HSV), notably type 1 (HSV-1), may constitute a risk factor of Alzheimer's disease (AD) [1], [2], [3], [4]. We recently assessed the association between seropositivity to HSV and risk of AD in the PAQUID study and found that elderly subjects who were IgM-positive were more likely to develop dementia within the next 7 years while no association was found among IgG-positive subjects [5]. These results suggest that a recent reactivation of HSV, characterized by the specific association with anti-HSV IgM antibodies, may be involved in the long-term neuropathological processes leading to dementia [5]. The identification of intermediary biomarkers within the amyloid cascade would considerably strengthen the case for the causal relationship suggested by epidemiological evidence. The relevance of plasma biomarkers of AD, notably of the amyloid β fragment (Aβ_1–40_ and Aβ_1–42_), has recently been investigated in humans, producing some conflicting results [6]. Indeed, in some studies, AD subjects exhibited higher Aβ_1–40_ or Aβ_1–42_ plasma levels compared to controls while others studies have reported opposing data [6]. We have previously reported that an increased Aβ_1–42_-to-Aβ_1–40_ plasma ratio was strongly negatively associated with the risk of dementia 2 years later in the Three-City population-based cohort, suggesting that changes in plasma Aβ_1–40_ and Aβ_1–42_ levels might be considered an indicator of short-term risk of dementia [7]. A meta-analysis of plasma Aβ levels in AD suggested that in longitudinal studies these parameters might be predictors of higher rates of progression to AD, and should be further explored as potential biomarkers [8].

In the relationship linking HSV to AD, our hypothesis is that a specific association between anti-HSV IgM, and not IgG, antibodies and plasma Aβ levels might occur in the pre-clinical phase of the dementia syndrome. The present study examined whether anti-HSV antibodies were associated with plasma Aβ_1–40,_ Aβ_1–42_ and Aβ_1–42_-to-Aβ_1–40_ ratio in a sample of older community dwellers from the Three-City study, and whether this association may be modulated by genetic risk factors for AD; Apolipoprotein E-allele e4 (ApoE4), *CR1* and *CLU*, which have also been involved in the HSV life cycle [2], [9].

## Results

The main study sample consisted of 1222 individuals, aged 73.9 y on average (range 65.0–94.1) and the secondary study sample, with *CLU*- and *CR1-* linked SNPs genotyping determination, consisted of 754 subjects, aged 74.0 y on average (range 65.0–92.0). Their main characteristics are described in **Table 1**.

**Table 1:** Main characteristics of the main study sample and the secondary study sample.

	Main study sample	Secondary study sample
	(n = 1222)	(n = 754)
Age, y, mean (SD)	73.9 (5.3)	74.0 (5.4)
Women, %	60.4	61.7
Low educational level,* %	60.6	59.1
ApoE e4-allele frequency, %	20.4	21.2
Aβ_1–42_, pg/mL, mean (SD)	38.9 (12.3)	39.1 (12.1)
Aβ_1–40_, pg/mL, mean (SD)	235.6 (66.2)	234.5 (63.8)
Aβ_1–42_/Aβ_1–40_ ratio, mean (SD)	0.17 (0.05)	0.17 (0.05)
IgG antibodies to HSV, IU/mL, mean (SD)	12.61 (7.03)	12.46 (7.11)
IgM antibodies to HSV, IU/mL, mean (SD)	0.053 (0.055)	0.052 (0.053)
*CR1* rs6656401† (%)	ND	34.0
*CR1* rs3818361† (%)	ND	33.2
*CLU* rs9331888‡ (%)	ND	48.4
*CLU* rs2279590• (%)	ND	66.7
*CLU* rs11136000• (%)	ND	64.7

Abbreviations: ApoE, apolipoprotein E; HSV, herpes simplex virus; Aβ, amyloid-beta; ND, not determined.

*Low educational level = short secondary school level or less.

† These genotyped markers of *CR1* were considered dichotomously: at least one adenine (GA or AA), the minor allele, vs. no adenine purine base (GG) in haplotypes.

‡ The genotyped marker of *CLU* rs9331888 was considered dichotomously: at least one guanine (CG or GG), the minor allele, vs. no guanine purine base (CC) in haplotypes.

• These genotyped markers of *CLU* were considered dichotomously: at least one thymine (TC or TT), the minor allele, vs. no thymine pyrimidine base (CC) in haplotypes. Eleven data for *CLU* rs11136000 were missing.

In the main study sample, only the crude correlation between anti-HSV IgM levels and plasma Aβ_1–40_ and Aβ_1–42_ levels were statistically significant (*r* = −0.074, *P* = 0.009 and *r* = −0.087, *P* = 0.002 respectively). Moreover, mean plasma Aβ_1–40_ and Aβ_1–42_ levels significantly differed according to the quartiles of anti-HSV IgM distribution in crude analyses (**Table 2**). The lowest mean plasma Aβ_1–40_ and Aβ_1–42_ levels were observed in the highest quartile of anti-HSV IgM. As a consequence, the mean Aβ_1–42_-to-Aβ_1–40_ ratio did not differ among quartiles of distribution of anti-HSV IgM. These results were virtually unchanged when these analyses were performed in the secondary study sample (n = 754) (**Table 3**). In contrast, there was no significant difference in means of plasma Aβ_1–40_ and Aβ_1–42_ and of the Aβ_1–42_-to-Aβ_1–40_ ratio according to quartiles of anti-HSV IgG distribution in the main study sample (**Table 2**) as in the secondary study sample (**Table 3**).

**Table 2:** Mean plasma amyloid-β levels by quartiles of distribution of IgM or IgG antibodies to herpes simplex virus in the main study sample (n = 1222).

*IgM antibodies to herpes simplex virus (IU/mL)*					
	1^st^ quartile	2^nd^ quartile	3^rd^ quartile	4^th^ quartile	
	≤0.018	[0.018–0.034]	[0.034–0.067]	>0.067	
Mean (SD)					P
Aβ_1–42,_ pg/mL	40.3 (12.7)	39.3 (13.2)	39.1 (12.2)	36.8 (10.7)	0.0036
Aβ_1–40_, pg/mL	241.4 (65.6)	243.2 (80.0)	230.3 (58.0)	226.9 (56.5)	0.0035
Aβ_1–42_-to-Aβ_1–40_ ratio	0.17 (0.04)	0.17 (0.05)	0.17 (0.05)	0.17 (0.06)	0.3566

*IgG antibodies to Herpes Simplex Virus (IU/mL)*					
	1^st^ quartile	2^nd^ quartile	3^rd^ quartile	4^th^ quartile	
	≤7.2	]7.2–15.0]	]15.0–18.0]	>18.0	
Mean (SD)					P
Aβ_1–42,_ pg/mL	38.29 (13.17)	40.04 (11.14)	38.56 (12.32)	38.42 (12.82)	0.2403
Aβ_1–40_, pg/mL	232.72 (66.61)	235.52 (58.65)	237.28 (69.11)	237.11 (71.91)	0.8195
Aβ_1–42_-to-Aβ_1–40_ ratio	0.17 (0.04)	0.18 (0.07)	0.17 (0.05)	0.17 (0.05)	0.0907

**Table 3:** Mean plasma amyloid-β levels by quartiles of distribution of IgM or IgG antibodies to herpes simplex virus in the secondary study sample (n = 754).

*IgM antibodies to herpes simplex virus (IU/mL)*					
	1^st^ quartile	2^nd^ quartile	3^rd^ quartile	4^th^ quartile	
	≤0.018	[0.018–0.033]	[0.033–0.066]	>0.066	
Mean (SD)					P
Aβ_1–42_, pg/mL	40.4 (11.7)	39.3 (13.0)	40.1 (13.0)	36.6 (10.3)	0.0097
Aβ_1–40_, pg/mL	242.5 (65.5)	240.4 (71.5)	229.8 (61.2)	225.0 (54.5)	0.0203
Aβ_1–42_/Aβ_1–40_ ratio	0.17 (0.04)	0.17 (0.06)	0.18 (0.05)	0.17 (0.06)	0.2401

*IgG antibodies to herpes simplex virus (IU/mL)*					
	1^st^ quartile	2^nd^ quartile	3^rd^ quartile	4^th^ quartile	
	≤6.90	[6.90–15.00]	[15.00–18.00]	>18.00	
Mean (SD)					P
Aβ_1–42_, pg/mL	39.0 (12.7)	40.1 (11.4)	38.0 (10.8)	39.2 (14.4)	0.3655
Aβ_1–40_, pg/mL	232.9 (66.1)	232.9 (56.7)	233.7 (54.4)	240.9 (83.5)	0.6575
Aβ_1–42_/Aβ_1–40_ ratio	0.17 (0.04)	0.18 (0.07)	0.17 (0.05)	0.17 (0.05)	0.2446

Associations between plasma Aβ_1–40_ and Aβ_1–42_ levels and of the Aβ_1–42_-to-Aβ_1–40_ ratio and anti-HSV IgM levels, considered as a continuous or class variable, in the main study sample are shown in **Table 4**. After adjustment for study center, age, sex and education, plasma Aβ_1–40_ and Aβ_1–42_ levels were significantly inversely associated with anti-HSV IgM (**Table 4** **, model 1**). The strength of these associations remained almost unchanged after additional adjustment for ApoE polymorphism (**Table 4** **, model 1+ApoE4**). When considering anti-HSV IgM levels as a class variable, the highest quartile of IgM was associated with a level of Aβ_1–42_ decreased on average of 2.9 pg/mL and a level of Aβ_1–40_ decreased of 11.6 pg/mL in fully adjusted models. No association between the plasma Aβ_1–42_-to-Aβ_1–40_ ratio and anti-HSV IgM levels was evidenced in multivariate linear regression analyses (**Table 4**). Considering anti-HSV IgG levels either as a continuous or a categorical variable, no association with plasma Aβ_1–40_, Aβ_1–42_ or the Aβ_1–42_-to-Aβ_1–40_ ratio were evidenced (**Table S1**). Finally, no significant statistical interaction with ApoE4 polymorphism was found in any model.

**Table 4:** Associations between plasma amyloid-ß levels and IgM antibodies to herpes simplex virus in the main study sample (n = 1222).

*IgM antibodies to herpes simplex virus*				
	Per one additional unit		4^th^ vs. 1^st^-2^nd^-3^rd^ quartiles	
	β (SE)	P	β (SE)	P
Aβ_1–42_				
Model 1	−20.9 (6.4)	0.001	−3.0 (0.8)	0.0003
Model 1+ApoE4*	−20.7 (6.4)	0.001	−2.9 (0.8)	0.0003
Aβ_1–40_				
Model 1	−93.0 (34.4)	0.007	−11.7 (4.4)	0.007
Model 1+ApoE4*	−92.4 (34.4)	0.007	−11.6 (4.4)	0.008
Aβ_1–42_/Aβ_1–40_ ratio				
Model 1	−0.0007 (0.03)	0.98	−0.002 (0.003)	0.56
Model 1+ApoE4*	0.0002 (0.03)	0.99	−0.002 (0.003)	0.57

Model 1 adjusted for study center, age, gender and educational level.

*Model 1 plus additional adjustment for apolipoprotein E-e4 polymorphism.

In a sensibility analysis, similar results were obtained when subjects who developed incident dementia during the follow-up were excluded (n = 40) (**Table S2**).

Given the potential involvement of the complement C3b protein, and so *CR1*, and of *CLU* in Aβ clearance and pathogen defence, associations between plasma Aβ_1–40_, Aβ_1–42_ levels and Aβ_1–42_-to-Aβ_1–40_ ratio and anti-HSV IgM or IgG levels were assessed in the secondary study sample where *CR1*- and *CLU*- linked SNPs markers were available. In this sub-population (n = 754), results of inverse associations between plasma Aβ_1–40_ and Aβ_1–42_ and anti-HSV IgM levels remained almost unchanged (**Table 5** **, model 1**). As previously observed in the main study sample, no association between the Aβ_1–42_-to-Aβ_1–40_ ratio and IgM antibody levels was found. Furthermore, no association between plasma Aβ_1–40_, Aβ_1–42_ and the Aβ_1–42_-to-Aβ_1–40_ ratio and anti-HSV IgG levels were observed (**Table S1**). When controlling for rs6656401 (**Table 5** **, model 1+** ***CR1***) or rs3818361 (**Table S3, model 1**) at the *CR1* locus, or for rs2279590 (**Table 5** **, model 1+** ***CR1***) or rs9331888 and rs11136000 (**Table S3, model 1 +** ***CR1*** **or** ***CLU***) at the *CLU* locus, results of inverse associations between plasma Aβ_1–40_ and Aβ_1–42_ and anti-HSV IgM were virtually unchanged. In fully adjusted models for ApoE4, *CR1-* and *CLU*- linked SNP, this inverse association remained significant (**Table 5**). No significant statistical interaction with any *CR1*- or *CLU*- linked SNP was found in any model.

**Table 5:** Associations between plasma amyloid-β levels and IgM antibodies to herpes simplex virus in the secondary study sample with *CR1*- and *CLU*-linked SNPs available data (n = 754).

*IgM antibodies to herpes simplex virus*				
	Per one additional unit		4^th^ vs. 1^st^-2^nd^-3^rd^ quartiles	
	β (SE)	P	β (SE)	P
Aβ_1–42_				
Model 1	−25.7 (8.3)	0.002	−3.5 (1.0)	0.0007
Model 1+*CR1* *	−25.6 (8.4)	0.002	−3.5 (1.0)	0.0008
Model 1+*CLU* †	−25.6 (8.3)	0.002	−3.5 (1.0)	0.0007
Model 1+*CR1*+*CLU* ‡	−25.5 (8.3)	0.002	−3.5 (1.0)	0.0007
Aβ_1–40_				
Model 1	−134.7 (43.6)	0.002	−12.0 (5.4)	0.03
Model 1+*CR1* *	−132.7 (43.6)	0.002	−11.6 (5.4)	0.03
Model 1+*CLU* †	−134.8 (43.6)	0.002	−11.9 (5.4)	0.03
Model 1+*CR1*+*CLU* ‡	−132.8 (43.7)	0.002	−11.6 (5.4)	0.03
Aβ_1–42_/Aβ_1–40_ ratio				
Model 1	0.02 (0.04)	0.61	−0.004 (0.005)	0.43
Model 1+*CR1* *	0.02 (0.04)	0.63	−0.004 (0.005)	0.41
Model 1+*CLU* †	0.02 (0.04)	0.60	−0.004 (0.004)	0.42
Model 1+*CR1*+*CLU* ‡	0.02 (0.04)	0.62	−0.004 (0.004)	0.40

Model 1 adjusted for study center, age, gender, educational level and apolipoprotein E-e4 polymorphism.

*Model 1 plus additional adjustment for *CR1* marker at rs6656401.

† Model 1 plus additional adjustment for *CLU* marker at rs2279590.

‡ Model 1 plus additional adjustment for *CR1* marker at rs6656401 and *CLU* marker at rs2279590.

## Discussion

This population-based cohort study is the first to report that higher plasma IgM antibodies to HSV levels were significantly associated with lower plasma Aβ_1–40_ and Aβ_1–42_ levels. No association between anti-HSV IgG antibodies and plasma Aß levels was highlighted. These results were independent of ApoE4 polymorphism, *CR1* and *CLU* markers.

Beside previous knowledge [5] [7], our hypothesis suggested that an association between anti-HSV IgM and plasma Aß levels would exist during the long prodromal phase of dementia. Although HSV was present in both normal and AD brains, several lines of evidence have already suggested potential scenarios by which HSV may participate in the complex pathogenesis of dementia [1], [3]. The brain areas which are predominantly targeted by HSV infectious agents in herpetic encephalitis include frontal cortex, temporal cortex and hippocampus, and are also those predominantly affected in AD [4]. Second, HSV-1 is ubiquitous and could reside latently in the central nervous system (CNS) or could easily enter the CNS because of a decline in the immune system with advancing age [10]. A hypothesis has suggested that periodic mild reactivation of the latent virus in the brain, because of age-related immunosuppression or stress, for the most part without evident clinical symptoms, may lead to increased cell damage, and indirectly, via inflammatory processes, increased susceptibility for AD [11]. This hypothesis has been in part confirmed in the PAQUID study [5] and altogether, these results were in favour of a long-term effect of recurrent reactivations of HSV leading to progressive brain damage, and several years later, to dementia. The replication of the PAQUID study analyses was not our main objective since participants of the case-cohort involved in the present analyses were followed-up only for 4 years.

The amyloid cascade hypothesis suggests that aberrant metabolism of the amyloid precursor protein (APP) and subsequent accumulation of oligomers Aß fragments is a major determinant of AD [12]. Repercussions of such brain alterations to peripheral Aß levels are only partly understood [6]. It has been suggested that plasma Aß levels gradually decreased over time with the increased brain Aß deposition in human as well [13]. Recent results of an increased PiB-PET uptake being associated with lower plasma Aß levels in Mild Cognitive Impairment supported the “sink hypothesis” that increased amyloid deposition in the brain is accompanied by lower peripheral Aß levels in plasma [14]. In that case, low plasma Aß levels might be considered possible short-term risk markers of dementia and could reflect prior sequestration of Aß in the brain [7], [13], [14], [15]. The finding by Yaffe et al. fitted comfortably within this hypothesis since plasma Aβ_1–42_ levels and the Aβ_1–42_-to-Aβ_1–40_ ratio correlated with cognitive decline [16]. Because of the fluctuation of plasma Aß levels during the presymptomatic dementia period, the accurate dynamic process of plasma Aß levels is not well known so far.

Interestingly, several studies have also linked HSV to Aß [11]. Indeed, a segment of Aß is highly homologous to a glycoprotein encoded by the virus, and an association between HSV-1 and APP during axonal transport of the virus may lead to alter the APP processing [11], [17], [18]. For instance, Cheng et al. have illustrated the possible role of HSV-1 in the APP dynamic, by showing that HSV-1-infected cells displayed abnormal APP distribution, and that APP and HSV-1 capsids co-localized and travelled together within cells [19]. Secondly, a striking localisation of HSV-1 DNA within amyloid plaques in human AD brains has also been reported [20]. Third, infection with HSV-1 increases the enzymes responsible for Aβ formation in mice brains and leads to Aβ accumulation [21], [22], [23]. In a neuronal cell culture model, HSV increases the formation of Aβ oligomers [24] while in HSV-1-infected cells, antiviral agents reduced the accumulation of Aβ and of phospho-Tau [25]. On the other hand, Aβ fibrils have been shown to facilitate the entry of several viruses, including HSV-1 [26], a mechanism considered as an initially protective response against the viral infection [4]. Anyway, an anti-infectious activity has been attributed to Aβ [27] suggesting that brain cells might produce Aβ in an attempt to combat infections.

Altogether, these results suggest that infection earlier in life with HSV in the peripheral nervous system, with subsequent infection in CNS, leads to activation of the immune system, altering APP metabolism. Recurrent periodic mild reactivation of latent HSV in CNS, as associated with anti-HSV IgM levels, may lead to gradually increased production of Aβ. The continuous Aβ accumulation in brains cells may eventually result in later changes in periphery as assessed by instability of plasma Aβ levels during the long prodromal phase of dementia and afterwards by decreased plasma Aβ levels in the very last years before the diagnosis [7], [15]. Toxicity-related Aβ over-production, concomitant with multiple HSV reactivations, thus in effect, a persistent infection, might be conducive to progressive brain injury, with increasing cognitive dysfunction, leading to dementia several years later [5]. If this hypothesis is true, the first HSV infection of brain may constitute a trigger for the amyloid cascade. The reactivation of the virus may exacerbate these brain alterations during the long time course preceding the clinical diagnosis of dementia. This is confirmed by unchanged results obtained by sensitivity analyses where subjects who developed dementia in the short-term (in the four subsequent years) were excluded in the present study. Finally, the lack of association between anti-HSV IgG antibodies, markers of past infection, and plasma Aß levels in the present study also reinforced the present hypothesis.

Inflammatory response of immune system against many viral infections, including HSV-1, is a possible indirect way by which this virus may contribute to AD [5], [11]. The exacerbation of neuroinflammation, due to HSV-1 infection and/or consequently to neuropathological processes, may contribute to increase oxidative stress, to which the CNS is highly sensitive [28]. Oxidative damage is commonly observed in AD, even in the early stages of the disease [29], and viral infection, such as HSV, also leads to over-production of reactive oxygen and nitrogen species [11], [30]. Finally, the efficacy of the autophagy, considered as usual cellular defences mechanism involved in AD, would be reduced by HSV-1 for its own survival [23], [31], [32], [33], [34].

Complex interactions between HSV life cycle and major susceptibility AD gene products, including *CR1* and *CLU* [9], have also recently been suggested [2], [11]. However, our results are not in favour of different associations between anti-HSV IgM and plasma Aβ among carriers of the *CR1* or *CLU* predisposing genetic factors. Associations between anti-HSV IgM and plasma Aβ levels were also not modulated by ApoE4 polymorphism in the present study, consistent with at least one previous observation that ApoE4 does not modify the association between HSV seropositivity and risk of AD [5], although other studies have suggested that it might [35], [36], [37]. Nevertheless, all potential genes/infection interactions have not yet been fully studied and require further investigation [2].

Our results should be interpreted with caution due to some limitations. First, since our study was cross-sectional, we could not determine whether the low observed plasma Aβ levels were the result of reactivation of HSV or whether plasma Aβ levels pre-dated elevated anti-HSV IgM levels. Indeed, an alternative explanation is that the possible accumulation of Aβ in brain cells, with subsequent low plasma Aβ levels, might be the first step of AD-related neuropathological processes, and might furthermore be characteristic of favourable conditions for latent HSV reactivation in the CNS. Second, plasma Aβ does not only reflect brain Aβ turnover and metabolism but also that derived from peripheral tissues [6], [38]. The relevance of repeated measurements of plasma Aβ and anti-HSV IgM levels to assess the timeline of the events during the prodromal period of the dementia process would reinforce our main hypothesis, although it was not obtainable. Moreover, measurement of plasma Aβ is subject to many potential confounds that induce biological variations and we can not exclude that these variations might in part increased our chance to evidence associations [39]. Cerebrospinal fluid (CSF) is thought to more closely reflect what is happening in the brain. CSF Aβ_1–42_ levels have been associated with current AD or shown to be predictive of future dementia in patients with Mild Cognitive Impairment [6]. Therefore, the replication of the present analyses with CSF biomarkers would be of great interest. No such samples were available in the 3C cohort, leading us to be unable to perform these analyses. However, among groups with different cognitive abilities in ADNI, the plasma Aβ had a better correlation with Aβ brain deposits than CSF Aβ values [40]. Third, the sub-type of HSV (HSV-1 or HSV-2) was not determined in this study, although it is most likely that participants were infected by HSV-1. Indeed, HSV-1 infection is more frequent than HSV-2 and herpes simplex encephalopathy caused by HSV-2 is very rare in adults [41].

Several strengths of this study must be underlined. As plasma Aβ concentration varies widely during the prodromal phase of dementia [7], our large sample size increases the validity of the observations. Moreover, this population-based study was conducted in the 3C cohort, which is independent sample of the previous cohort (the PAQUID study) [5].

To conclude, we have shown that HSV reactivation, assessed by increased anti-HSV IgM levels, is associated with lower plasma Aβ_1–40_ and Aβ_1–42_ levels, lending further support to the hypothesis that HSV may be implicated in the dynamic of the APP processing and potentially in the pathogenesis of AD in human. Further research is needed to establish the direction of causality and to explain the underlying mechanisms.

## Methods

### Participants

The data come from the Three-City (3C) study, a prospective cohort study of vascular risk factors of dementia whose methodology is described in detail elsewhere [42]. The protocol of the 3C study was approved by the Consultative Committee for the Protection of Persons participating in Biomedical Research of the Kremlin-Bicêtre University Hospital (Paris). A sample of 9294 community dwellers aged 65 and over was selected in 1999–2000 from the electoral rolls of three French cities: Bordeaux (n = 2104), Dijon (n = 4931) and Montpellier (n = 2259). All participants signed a written consent and all clinical investigations have been conducted according to the principles expressed in the “Declaration of Helsinki”.

At the baseline clinical examination, data were assessed using standardised questionnaires and a blood sample was obtained. Participants were reexamined two (2001–2003; n = 8072) and four (2003–2005; n = 7148) years after the baseline examination. During this follow-up period, incident dementia were actively screened, using a two step procedure following administration of the battery of neuropsychological tests [42]. At each wave, participants suspected of having dementia based on their present neuropsychological performances or decline relative to a previous examination were examined by a neurologist. An independent committee of neurologists then reviewed all potential cases of dementia and analysed in depth the medical history of each participant to obtain a consensus on the diagnosis and etiology according to the criteria of the *Diagnostic and Statistical Manual of Mental Disorders*, fourth edition.

**Figure 1:**
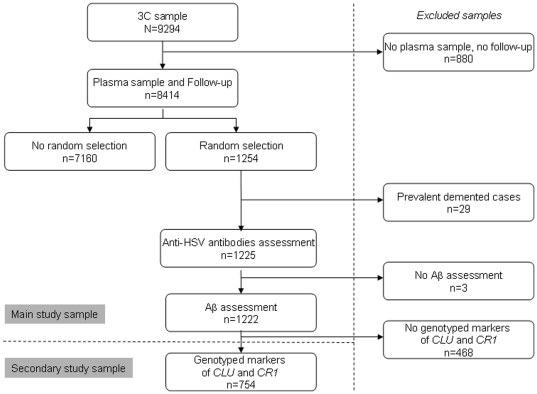
Design of the case-control study, definition of the main study sample and of the secondary study sample. Abbreviations: HSV, herpes simplex virus; Aβ, Amyloid-beta; 3C, Three-City Study.

A case–cohort study was conducted at the end of 4 years of follow-up for the investigation of non-standard risk markers for dementia, stroke and coronary heart disease (**Fig. 1**). Among the 9294 subjects of the initial cohort, 880 were excluded because either they had no blood sampling or they did not participate in any of the follow-up examinations, leading to a remaining sample of 8414. For the present work, the case–cohort study comprised a subcohort of 1254 subjects randomly selected in strata defined according to center, age (5 years), and sex. Among them, twenty-nine subjects were diagnosed as having prevalent dementia at baseline and were thus excluded from the current analysis. Incident dementia was diagnosed in 40 participants included in the subcohort. Participants for whom at least one Aß plasma concentration (n = 3) or IgM or IgG antibodies to HSV quantification (n = 0) was missing were excluded. These selection steps allowed us to define a main study sample of 1222 participants (**Fig. 1**).

### Assessment of plasma amyloid-β concentration

Blood samples were all obtained early in the morning, simultaneously to the baseline data collection. Blood was collected in anticoagulant (EDTA) vacutainers and centrifuged at 1.000 g for 10 minutes. Plasma samples were aliquoted and were frozen immediately at −80°C.

The plasma Aβ quantification was described in details elsewhere [7]. Briefly, the baseline plasma Aβ peptide assay was performed using an INNO-BIA kit (Innogenetics, Ghent, Belgium) based on a multiplex xMAP (Luminex, Austin, TX) technique. Knowing the dynamic of plasma amyloid levels according to matrix type and technical processing, we used the INNO-BIA kit as one of the more reliable commercial amyloid ELISA kits [39]. The quantification of Aβ_1–40_ and Aβ_1–42_ (pg/mL) were determined and the Aβ_1–42_-to-Aβ_1–40_ ratio was computed.

### IgM and IgG antibodies to Herpes Simplex Virus quantification

A high sensitive and specific ELISA diagnostic kit (Enzygnost Anti HSV/IgM and IgG, Dade Behring, Marburg, Germany) was used to quantify anti-HSV IgM antibodies (anti-HSV IgM) and anti-HSV IgG antibodies (anti-HSV IgG) [5], [43]. IgM and IgG titres are expressed in international unit per milliliter (UI/mL).

### Potential confounders

Socio-demographic information included age, sex, and education. Apolipoprotein E (ApoE) genotyping was performed at the Lille Genopole (France) and ApoE4 genotype was considered dichotomously: presence of at least one e4 allele vs. no e4 allele [44]. DNA of a subsample of participants of the 3C study, transferred to the French Centre National de Genotypage for genome wide assessment, gives us information on *CLU*- and *CR1*-linked SNPs genotyping [9]. Among them, 754 subjects of the case-control study, for whom markers of *CR1-*linked SNPs (rs6656401 and rs3818361) and *CLU*-linked SNPs (rs9331888, rs2279590 and rs11136000) have been determined, constituted the secondary study sample for the present analysis (**Fig. 1**) [9]. Eleven data for *CLU* rs11136000 were missing.

### Statistical analyses

All statistical analyses were performed with SAS Statistical package (Version 9.1 SAS Institute). Demographic, biological and genetic characteristics were described in the main study sample (n = 1222) and in the secondary study sample (n = 754). In the main study sample, the crude association between plasma Aβ_1–40_, Aβ_1–42_ and the Aβ_1–42_-to-Aβ_1–40_ ratio and the anti-HSV IgM or anti-HSV IgG levels were performed. Moreover, the quartiles of distribution of anti-HSV IgM and anti-HSV IgG were defined and mean plasma Aβ_1–40_, Aβ_1–42_ and the Aβ_1–42_-to-Aβ_1–40_ ratio were compared using analysis of variance (ANOVA) or Kruskal-Wallis test when ANOVA hypotheses were not satisfied (accepted significance at *P*<0.05). Cross-sectional analyses of the association between plasma Aβ_1–40_ and Aβ_1–42_ levels and the Aβ_1–42_-to-Aβ_1–40_ ratio (entered into separate models as continuous variables) and anti-HSV IgM or anti-HSV IgG were separately performed by multivariate linear regression. Anti-HSV IgM or IgG levels have been considered as continuous variable on the one hand (i.e. analysis for one additional unit of IgM or IgG) and as dichotomous variable on the other hand: the highest quartile of distribution of anti-HSV IgM or IgG was compared with the clustered three other quartiles, chosen as reference. These analyses were adjusted for study center, age (continuous), sex, and education level in model 1 and additionally for ApoE genotype in model 2. Statistical interactions between IgM or IgG levels and ApoE genotype were tested. In a sensitivity analysis, subjects with incident dementia (n = 40) were excluded.

All these analyses were replicated in a sub-sample of 754 subjects with available data on genotyped markers of *CR1* and *CLU* (**Fig. 1**). Multivariate linear regression models of the association between plasma Aβ_1–40_, Aβ_1–42_ levels and the Aβ_1–42_-to-Aβ_1–40_ ratio (entered into separate models as continuous variables) and anti-HSV IgM or IgG were adjusted for study center, age (continuous), sex, education level and ApoE genotype in model 1. Additional adjustments for *CR1* markers (rs6656401 on the one hand and rs3818361 on the other hand) and for *CLU* markers (rs9331888, rs2279590 and rs11136000 in separated models) were performed. Finally, additional models taken into account the ApoE4 genotype, *CR1* and *CLU* markers as adjustment variables have been performed. Statistical interactions between IgM or IgG levels and *CR1*- or *CLU-* linked SNPs were tested.

## Supporting Information

Table S1 Associations between plasma amyloid-β levels and IgG antibodies to herpes simplex virus in the main study sample (n = 1222) and in the secondary study sample with *CR1*- and *CLU*-linked SNPs available data (n = 754).
(DOC)

Table S2 Associations between plasma amyloid-β levels and IgM and IgG antibodies to herpes simplex virus in subjects from the main study sample who remained free from dementia over time (n = 1182).
(DOC)

Table S3 Associations between plasma amyloid-β levels and IgM antibodies to Herpes Simplex Virus in the secondary study sample with *CR1*- and *CLU*-linked SNPs available data (n = 754).
(DOC)
