# Supplementary material for: Association between IgM Anti-Herpes Simplex Virus and Plasma Amyloid-Beta Levels
Source: PLoS One. 2011 Dec 28;6(12):e29480. doi: 10.1371/journal.pone.0029480 (PMC3247269; doi:10.1371/journal.pone.0029480)
Supplement: Table S1 — Associations between plasma amyloid-β levels and IgG antibodies to herpes simplex virus in the main study sample (n = 1222) and in the secondary study sample with CR1- and CLU-linked SNPs available data (n = 754). (DOC) [file pone.0029480.s001.doc]

**Table S1.** Associations between plasma amyloid-β levels and IgG antibodies to herpes simplex virus in the main study sample (n=1222) and in the secondary study sample with *CR1*- and *CLU*-linked SNPs available data (n=754)

| *Main study sample: IgG antibodies to herpes simplex virus* | | | | | |
| --- | --- | --- | --- | --- | --- |
|  | Per one additional unit |  |  | 4^th^ vs. 1^st^-2^nd^-3^rd^ quartiles |  |
|  | β (SE) | P |  | β (SE) | P |
| Aβ_1–42*_ | -0.0009 (0.05) | 0.98 |  | -0.873 (0.941) | 0.35 |
| Aβ_1–40*_ | 0.304 (0.274) | 0.27 |  | 2.10 (5.04) | 0.68 |
| Aβ_1–42_/Aβ_1–40_ ratio* | -0.0001 (0.0002) | 0.52 |  | -0.0066 (0.0039) | 0.09 |
| *Secondary study sample: IgG antibodies to herpes simplex virus* | | | | | |
|  | Per one additional unit |  |  | 4^th^ vs. 1^st^-2^nd^-3^rd^ quartiles |  |
|  | β  (SE) | P |  | β (SE) | P |
| Aβ_1–42*_ | -0.013 (0.063) | 0.84 |  | -0.342 (1.18) | 0.77 |
| Aβ_1–40*_ | 0.334 (0.331) | 0.31 |  | 8.62 (6.17) | 0.16 |
| Aβ_1–42_/Aβ_1–40_ ratio* | -0.0001 (0.0002) | 0.57 |  | -0.010 (0.005) | 0.06 |

*Results are adjusted for study center, age, gender, educational level and apolipoprotein E-ε4 status
