# Supplementary material for: Association between IgM Anti-Herpes Simplex Virus and Plasma Amyloid-Beta Levels
Source: PLoS One. 2011 Dec 28;6(12):e29480. doi: 10.1371/journal.pone.0029480 (PMC3247269; doi:10.1371/journal.pone.0029480)
Supplement: Table S2 — Associations between plasma amyloid-β levels and IgM and IgG antibodies to herpes simplex virus in subjects from the main study sample who remained free from dementia over time (n = 1182). (DOC) [file pone.0029480.s002.doc]

**Table S2.** Associations between plasma amyloid-β levels and IgM and IgG antibodies to herpes simplex virus in subjects from the main study sample who remained free from dementia over time (n=1182)

| *IgM antibodies to herpes simplex virus* | | | | | |
| --- | --- | --- | --- | --- | --- |
|  | Per one additional unit |  |  | 4^th^ vs. 1^st^-2^nd^-3^rd^ quartiles |  |
|  | β (SE) | P |  | β (SE) | P |
| Aβ_1–42*_ | -19.08 (6.40) | 0.003 |  | -2.77 (0.82) | 0.0007 |
| Aβ_1–40*_ | -75.80 (33.67) | 0.025 |  | -9.93 (4.31) | 0.021 |
| Aβ_1–42_/Aβ_1–40_ ratio* | -0.005 (0.027) | 0.84 |  | -0.002 (0.003) | 0.477 |
| *IgG antibodies to herpes simplex virus* | | | | | |
|  | Per one additional unit |  |  | 4^th^ vs. 1^st^-2^nd^-3^rd^ quartiles |  |
|  | β (SE) | P |  | β (SE) | P |
| Aβ_1–42*_ | -0.014 (0.051) | 0.78 |  | -1.25 (0.95) | 0.19 |
| Aβ_1–40*_ | 0.269 (0.27) | 0.32 |  | 0.27 (4.99) | 0.96 |
| Aβ_1–42_/Aβ_1–40_ ratio* | -0.0002 (0.0002) | 0.41 |  | -0.007 (0.004) | 0.063 |

*Results are adjusted for study center, age, gender, educational level and apolipoprotein E-e4 polymorphism
