# Supplementary material for: Association between IgM Anti-Herpes Simplex Virus and Plasma Amyloid-Beta Levels
Source: PLoS One. 2011 Dec 28;6(12):e29480. doi: 10.1371/journal.pone.0029480 (PMC3247269; doi:10.1371/journal.pone.0029480)
Supplement: Table S3 — Associations between plasma amyloid-β levels and IgM antibodies to Herpes Simplex Virus in the secondary study sample with CR1- and CLU-linked SNPs available data (n = 754). (DOC) [file pone.0029480.s003.doc]

**Table S3.** Associations between plasma amyloid-β levels and IgM antibodies to Herpes Simplex Virus in the secondary study sample with *CR1*- and *CLU*-linked SNPs available data (n=754)

| *IgM antibodies to herpes simplex virus* | | | | | |
| --- | --- | --- | --- | --- | --- |
|  | Per one additional unit |  |  | 4^th^ vs. 1^st^-2^nd^-3^rd^ quartiles |  |
|  | β (SE) | P |  | β (SE) | P |
| Aβ_1–42_ |  |  |  |  |  |
| Model 1+ *CR1* | -25.8 (8.4) | 0.002 |  | -3.5 (1.0) | 0.0007 |
| Model 1+ *CLU** | -25.7 (8.3) | 0.002 |  | -3.5 (1.0) | 0.0007 |
| Model 1+ *CLU*^†^ | -24.4 (8.4) | 0.004 |  | -3.3 (1.0) | 0.001 |
|  |  |  |  |  |  |
| Aβ_1–40_ |  |  |  |  |  |
| Model 1+ *CR1* | -132.6 (43.7) | 0.002 |  | -11.6 (5.4) | 0.03 |
| Model 1+ *CLU** | -133.1 (43.6) | 0.002 |  | -11.8 (5.4) | 0.03 |
| Model 1+ *CLU*^†^ | -127.8 (43.8) | 0.004 |  | -11.1 (5.4) | 0.04 |
|  |  |  |  |  |  |
| Aβ_1–42_/Aβ_1–40_ ratio |  |  |  |  |  |
| Model 1+ *CR1* | 0.02 (0.04) | 0.65 |  | -0.004 (0.005) | 0.38 |
| Model 1+ *CLU** | 0.02 (0.03) | 0.66 |  | -0.004 (0.004) | 0.39 |
| Model 1+ *CLU*^†^ | 0.01 (0.03) | 0.85 |  | -0.006 (0.004) | 0.19 |

Model 1 adjusted for study center, age, gender, educational level, apolipoprotein E-e4 status and *CR1* marker at rs3818361

*Model adjusted for study center, age, gender, educational level, apolipoprotein E- e 4 status and *CLU* marker at rs9331888

^†^ Model adjusted for study center, age, gender, educational level, apolipoprotein E- e 4 status and *CLU* marker at rs11136000. Eleven data for *CLU* rs11136000 were missing (n=743).
